# Trajectories of Pain in Very Old Age: The Role of Eudaimonic Wellbeing and Personality

**DOI:** 10.3389/fpain.2022.807179

**Published:** 2022-02-24

**Authors:** Markus Wettstein, Oliver Karl Schilling, Hans-Werner Wahl

**Affiliations:** ^1^Department of Psychology, Humboldt-Universität zu Berlin, Berlin, Germany; ^2^Network Aging Research, Heidelberg University, Heidelberg, Germany; ^3^Department of Psychological Aging Research, Psychological Institute, Heidelberg University, Heidelberg, Germany

**Keywords:** neuroticism, extraversion, terminal decline, purpose in life, autonomy, primary aging, tertiary aging, biopsychosocial model of pain

## Abstract

Pain is common in very old age and in the last years prior to death. However, little is known regarding longitudinal trajectories of pain in very old age and at the end of life. Moreover, whereas medical and morbidity-related factors contributing to pain are established, the role of psychosocial factors, such as eudaimonic wellbeing or personality as potential determinants of late-life pain trajectories has so far not been sufficiently investigated. We used data from the LateLine project. The sample consisted of *n* = 118 very old adults (*M* = 90.5 years, *SD* = 2.8 years) who were living alone at baseline and who had died between 2009 and 2021. They took part in up to 16 measurement occasions (*M* = 5.2, *SD* = 4.7, range 1–16) within an observational interval of 7 years. Assessment of pain was based on the SF-36 bodily pain subscale. Key indicators of eudaimonic wellbeing (autonomy, environmental mastery, and purpose in life) as well two of the Big Five personality traits (neuroticism and extraversion) were included as predictors. We controlled in all analyses for gender, education, subjective health, and depressive symptoms. Contrasting pain trajectories over chronological age (time since birth) vs. time to death, a time-to-death-related model resulted in a better model fit and accounted for a larger amount of pain variability than the age-related model. Mean-level change in pain, both over age and time to death, was not significant, but there was substantial interindividual variability in intraindividual trajectories. Age-related change in pain was significantly predicted by autonomy and neuroticism, with increasing pain among those who had lower initial autonomy scores and higher initial neuroticism scores. With regard to time-to-death-related trajectories of pain, higher purpose in life as well as lower extraversion at baseline predicted less increase or even steeper decrease in pain with approaching death. Our findings suggest that, despite overall mean-level stability in pain both over age and time to death, there is a substantial proportion of individuals who reveal deterioration in pain over time. Regarding the role of psychosocial predictors, personality traits and eudaimonic wellbeing are related with late-life pain trajectories both over age and time-to-death.

## Trajectories of Pain in Very Old Age: The Role of Eudaimonic Wellbeing and Personality

Pain is a common condition in old and very old age (1–5), and it is even the most frequent health symptom among older adults (6). Based on a sample of Swedish oldest-old, Zarit et al. (7) reported a pain prevalence of 34%, which increased to 40% after 2 years. According to Zimmer and Rubin (6), “more than one-half of older people at any point in time are experiencing pain” (p. 220). Moreover, pain is also common in the last years and months prior to death (8).

The detrimental consequences of pain for developmental outcomes such as quality of life and mental health (9–11), functional ability (12–14) or longevity (15, 16) are well-established from prior research. Yet, little is known with regard to long-term trajectories of pain among the oldest-old as well as with regard to their predictors. This is particularly true with regard to psychosocial factors, whose role for the onset or progression of pain specifically in advanced old age has, unlike the role of biological and morbidity-related factors, so far found only very limited empirical attention. Pain seems to be a “silent epidemic” in old age (1), as it is often not sufficiently treated based on pharmacological as well as non-pharmacologic therapies among older and very old adults (3, 4). Old and very old adults are often excluded from clinical trials on pain [e.g., (1, 17)], and they are at a heightened risk for inadequate pain treatment (18). For instance, Miaskowski et al. (4) state that “for older adults, access to non-pharmacologic therapies is limited because these types of interventions are expensive, often not recommended by clinicians, or not available in the community.” This exclusion of older adults from pain trials, but also from pain treatment further contributes to missing evidence regarding pain in (very) old age, its plasticity, its determinants, and its treatment.

The goal of this study is therefore to investigate trajectories of pain among the oldest-old, using a longitudinal data set comprising a 7-year assessment period. As most study participants had died between 2009 and 2021, we will examine and compare trajectories of pain over chronological age (time since birth) vs. over time-to-death. Finally, given the already mentioned lack of research on psychosocial determinants of pain, we will also investigate the role of crucial psychosocial factors, i.e., eudaimonic wellbeing as well as major personality traits (neuroticism, extraversion), for pain trajectories in advanced old age.

### Characteristics of Very Old Age

Very old age, or the “4th age” (19, 20), begins—according to a common population-based definition (19)—when 50% of one's birth cohort are no longer alive. The transition into very old age in developed countries might thus occur in the 8th decade of life. The fourth age is a peculiar life phase that is characterized by a pronounced ambiguity: On the one hand, physical vulnerability and experiences of loss, e.g., in the domains of cognitive functioning and sensory abilities, accumulate in very old age; on the other hand, very old adults represent a selective group of survivors who have outlived many of their peers, possibly due to exceptional resources. This ambiguity is also reflected by discrepant trends in subjective vs. objective health in very old age. Specifically, most oldest-old report that they are satisfied with their health [and their lives; (21, 22)] and reveal rather stable patterns—or among some individuals even improvements (23–25)—of subjective health indicators over time (26). In contrast, indicators of objective health are compromised in very old age and reveal a consistent pattern of pronounced decline (21, 26, 27). Late-life declines in objective health are particularly steep when the very last years of life are considered from a time-to-death-related change perspective, as pronounced dynamics of “terminal decline” in wellbeing and other domains such as functional or cognitive ability have been observed with increasing proximity to death (28–30).

### Pain Trajectories in Very Old Age

Regarding pain in very old age, it is important to point out that no clear “pain biomarker” exists, so that a substantial extent of subjectivity is involved whenever pain is reported [(6), p. 219]. In very old age, these self-reports might be affected by some tendency toward positive perceptions, potentially reflecting the so-called “age-related positivity effect” (31) and similar to very old adults' favorable self-perceptions of health (21, 26, 27). That is, by adjusting their standards accordingly [“response shift”; (32)] and using downward comparisons (33, 34), very old adults might report low levels of pain even when affected by the experience of (chronic) pain. They might assume that most of their peers are not pain-free and that pain is thus to some extent a natural part of growing older (4) and—other than at younger ages—a to some extent anticipated experience of old and very old age [(35), p. 274: “When you're this age, and you have an ache, so what? You expect to have aches when you're this age”]. In line with this assumption, Zarit et al. (7) observed associations of only small effect size between pain in the oldest-old and domains such as subjective health, depressive symptoms, or mobility and interpret this as evidence in support of “adaptation and selectivity among survivors in very late life” (p. 459).

With regard to general developmental dynamics in late life, Birren and Cunningham (36) distinguished between processes of tertiary aging—characterized by “accelerated functional deteriorations that manifest shortly (months, maybe years) before death” [(37), p. 28]—and those of primary and secondary aging. Whereas, secondary (or pathological) aging refers to changes that do not occur age-graded, but due to disease and/or disability, and that might be preventable or reversible, primary aging refers to “normative” aging, i.e., changes that unfold over chronological age due to biological or physical decline [e.g., (30)]. Thus, apart from non-normative secondary aging processes that cause pain (e.g., diseases), “typical” developmental changes in the prevalence of pain in late life may unfold not only in association with advancing chronological age, but also—and maybe even to a stronger extent—with individuals' shortening time-to-death, i.e., as part of tertiary aging. Indeed, such time-to-death-related “terminal increase” in pain with advancing proximity to death seems plausible. Findings of terminal decline across various developmental domains, including wellbeing, cognitive functioning, functional ability, and health (28–30, 38–41), suggest that self-regulatory capacities indeed get increasingly depleted with advancing proximity to death (42). Such depletion might in turn also affect individuals' capabilities to maintain favorable and stable pain perceptions, consequently resulting in an increase of pain with increasing proximity to death. In line with this assumption, previous research found that pain is quite common prior to death (8), with pain prevalence increasing among those who have reached their last 4 months of life (43).

Altogether, empirical examinations of late-life changes limited to a solely age-related perspective may thus miss a crucial part of the developmental dynamics, overestimate late-life stability and underestimate late-life decline, whenever change is more pronounced with increasing proximity to death than with increasing age (30, 37, 44). If so, incorporating the time-to-death perspective is crucial to provide a more clear-cut and complete picture of the changes that unfold in individuals' final years of life. Particularly in very old age, a life phase that is, by definition, characterized by a relative closeness to death, taking a time-to-death-related perspective on developmental changes, in addition to an age-related perspective, seems to be important. In this study, we will therefore consider pain trajectories in very old age from both time perspectives, investigating and contrasting within-person changes in pain both over chronological age as well over time-to-death.

### Psychosocial Factors Associated With Pain in Very Old Age: Conceptual Considerations

Pain in later life may be seen as a prototypical case for the interaction among biological, social, and psychological factors as determinants of adult development and aging [see (45)]. Consequently, the biopsychosocial model of chronic pain (3, 4, 46) posits that the onset, progression, and individual experience of pain should be seen as an outcome of all three components. Of note, the “hallmark of the biopsychosocial model of pain and its management is the notion that pain is a complex experience that is influenced not only by its underlying pathophysiology” [(3); p. 418], but by various factors beyond morbidity and physiology such as psychosocial determinants [see also (4)]. The biopsychosocial model of chronic pain also reveals some conceptual overlap with the established disablement process model (47, 48). This model is one of the most essential and established conceptual efforts in gerontology and geriatric medicine to combine biomedical, behavioral, and intervention-related and rehabilitative perspectives. It postulates that whether and to what extent risk factors (such as pain) result in functional limitations and disability depends on various factors, including intra-individual factors such as psychosocial attributes. Wellbeing and personality are key examples of such psychosocial attributes and are therefore focused in this study. Important for the consideration of pain in very old age, both wellbeing and personality, are—in analogy to pain—susceptible to age-related changes [e.g., (22, 49, 50)] as well as to time-to-death-related changes [e.g., (28, 51)]. Late-life changes in components of pain (such as pain magnitude or pain interference) could thus to some extent be driven by concurrent changes in wellbeing and personality. With regard to practical implication, such psychosocial determinants are—unlike genetic or other factors—to some extent modifiable and could thus be implemented in holistic, multi-component approaches of pain prevention or treatment.

### The Role of Wellbeing for Pain in Advanced Old Age

Wellbeing is a multidimensional construct [e.g., (52)] comprising multiple domains. An established theoretical distinction is the one between hedonic vs. eudaimonic wellbeing (53–55). Whereas, hedonic wellbeing refers to happiness, pursuit of pleasure and avoidance of pain, eudaimonic wellbeing refers to aspects of meaning, self-realization, and basic needs that should be fulfilled to achieve a “good” (late) life.

Particularly the fulfillment - or lacking fulfillment - of eudaimonic wellbeing and respective needs might be an important resource for coping with and adjusting to pain in very late life. Eudaimonic indicators have indeed been found to predict health, disability and mortality (56–61), they are associated with various biological correlates, including daily salivary cortisol, pro-inflammatory cytokines, cardiovascular risk, and REM sleep duration (62). Eudaimonic wellbeing might thus be a factor that promotes physiological functioning (63), and it could also be an important compensatory psychosocial resource that prevents or buffers pain and minimizes the impact on pain on everyday life functioning. Also, eudaimonic wellbeing components could be motivating factors that make individuals seek help and treatment when pain sets in instead of feeling helpless, giving up and adopting a fatalistic attitude.

We focus on three eudaimonic domains which might play an important role for pain trajectories in very late life, namely autonomy, environmental mastery, and purpose in life. All three indicators are positively related with each other [e.g., (64)]. Experiencing relatively high autonomy and environmental mastery may act as resources for adjustment to pain, as they represent—according to self-determination theory (53)—two basic psychological needs (autonomy and competence). In addition, having a purpose in life might also contribute to accumulating psychosocial resources that are helpful for coping with the adversity of pain in the situation of very old age and impending death. Indeed, purpose in life, or—more generally—meaning in life “is linked concurrently and prospectively with a huge range of desirable psychological and physical outcomes” [(65); p. 382]. For instance, individuals with a greater purpose in life reveal a better social integration and relational quality (66), which might be an important resource to prevent or cope with pain. A sense of purpose in life also enables individuals to set and pursue goals and to show active life engagement (67), which might also be an adaptive coping strategy when pain sets in. Additionally, a greater purpose in life has been found to be protective against cognitive decline and impairment (68, 69), and lower cognitive functioning is in turn a meaningful predictor of chronic pain (70).

With regard to associations of these eudaimonic indicators with age and time-to-death, environmental mastery and purpose in life have been reported to be negatively related with age and to decrease in old and very old age, whereas autonomy is not systematically related with age and remains stable in very old age (22, 66, 69, 71, 72).

### The Role of Personality for Pain in Advanced Old Age

Personality traits, most often operationalized by the Big Five personality traits (73–75), are, *via* behavioral—e.g., health behaviors such as smoking (76, 77)—and other pathways, important determinants of health (67, 78) and mortality (77). Of note, personality and wellbeing are interrelated; purpose in life, for instance, is positively associated with extraversion and negatively related with neuroticism (67, 79).

Personality traits are also related with pain. For instance, pain patients' personality profiles significantly deviate from population-based normative scores or from pain-free control groups (80–82). In this study, we will focus on two of the Big Five traits, namely neuroticism and extraversion. Whereas, higher neuroticism is associated with worse health outcomes, such as poorer self-rated (83–85) or physician-rated health (86, 87), associations of extraversion with health outcomes are positive (88–90).

These two traits, neuroticism and extraversion, were also found to have an impact on how individuals react to and cope with stressors—and pain might be a major stressor for many very old adults. Specifically, higher neuroticism is generally associated with a higher reactivity to stressors (91) and with use of passive and ineffective coping strategies (92). Higher neuroticism is also positively associated with reporting physical symptoms (93) and seems to lower the threshold from which on an individual perceives pain as threatening (94). High neuroticism might complicate the adjustment to health conditions and rather augment their negative consequences. For instance, among those older individuals who score higher on neuroticism, sensory impairments are more closely associated with lower cognitive abilities and higher risk of cognitive decline (95, 96) as well as of functional ability decline (97). Also, the association of pain, e.g., with less favorable self-evaluations of health, seems to be more negative among individuals who score higher on neuroticism (83). In contrast, higher extraversion is related with active coping strategies (98), such as social support seeking, problem-focused coping or positive reappraisal (92, 99). However, the potential role of personality in the life phase of very old age for outcomes of health and functioning in general, including pain, is not well-understood so far (100).

Similar to pain and eudaimonic wellbeing, personality traits are also subject to change in very old age and with increasing proximity to death. Specifically, neuroticism increases in (very) old age (49) and particularly at the end of life (51), whereas extraversion decreases late in life (50, 51).

### The Present Study

In this study, we investigate pain trajectories among the oldest-old. Our research goals are:

1. to analyze mean-level trajectories of pain in a sample of very old individuals as well as interindividual variability in within-person changes both over time-since-birth (age) and over time-to-death. We make use of an intensive data-collection design that included up to 16 measurement occasions over a 7-year period.
2. to investigate the role of psychosocial determinants, namely eudaimonic wellbeing (autonomy, environmental mastery, and purpose in life) as well as personality (neuroticism and extraversion), for pain trajectories in advanced old age, again both over calendar age and over time-to-death. We assume that eudaimonic wellbeing indicators are psychosocial resources that prevent or buffer increases in pain, both over age and over time-to-death. We also expect that unfavorable personality traits—particularly high neuroticism scores—are associated with an increase in pain among the oldest-old, both across age and across time-to-death. As part of exploratory analyses, we will compare the predictive role of eudaimonic wellbeing and personality for age-related vs. for time-to-death-related pain trajectories, as the strength of predictors in general can be different according to whether chronological age or time-to-death is considered [e.g., (101)].

## Materials and Methods

We used data from the longitudinal project LateLine (22, 26, 102, 103). This study project comprises up to 16 measurement occasions that took place between 2009 and 2016 (T1–T16). The LateLine study followed up a German random sample (*n* = 124) of originally 450 older individuals that had been drawn in 2002 as part of another study ENABLE-AGE project; detailed information on this parent sample and its recruitment are reported by Iwarsson et al. (104). Study participants were originally living alone in the Heidelberg-Mannheim area, and they were born between 1912 and 1922. The sample for the present analyses consists of *n* = 118 very old individuals who had died between 2009 and 2021 (two individuals were still alive in September 2021; status of four individuals could not be determined). Dates of death were obtained *via* information of relatives or of city registries. Overall, participants' age across all measurement occasions ranged from 87 to 102 years. Time to death across all measurement occasions ranged from 0 to 148 months (0 indicating that the observation took part within the participant's last month prior to death). Each study participant provided, on average, 5.2 observations (*SD* = 4.7, range 1–16; individuals with one observation: 33 (28.0%); two observations: 19 (16.1%); three observations: 10 (8.5%); four observations: 6 (5.1%); five observations: 5 (4.2%); six observations: 9 (7.6%); seven observations: 5 (4.2%); eight observations: 5 (4.2%); nine observations: 1 (0.8%); 10 observations: 4 (3.4%); 11 observations: 4 (3.4%); 12 observations: 5 (4.2%); 13 observations 2 (1.7%); 14 observations: 0 (0%); 15 observations: 4 (3.4%); 16 observations: 6 (5.1%).

Data collection was carried out by trained interviewers during home visits. Participants with probable severe cognitive impairment, i.e., with a score <17 on the Mini-Mental State examination [MMSE; (105)] were excluded from study participation. A description of the study sample and of the intercorrelations between study variables is provided in Table 1. Years of education (including school and higher-education institutions such as universities) ranged from 9 to 18 years, with a mean of ~12.5 years.

**Table 1:** Means, standard deviations, and intercorrelations of study variables at baseline (T1, 2009).

	***M* or *n***	***SD* or %**										
			**2**	**3**	**4**	**5**	**6**	**7**	**8**	**9**	**10**	**11**
1. Pain^a^ (0–100)	64.03	25.88	0.04	0.26**	0.12	−0.32**	−0.44***	0.11	0.16	−0.17	−0.01	0.17
2. Autonomy (1–5)	3.98	0.55		0.20*	−0.07	−0.11	−0.03	−0.14	0.10	−0.10	0.00	−0.06
3. Environmental mastery (1–5)	4.07	0.57			0.31**	−0.61***	−0.34***	−0.17	0.15	−0.09	−0.30	0.23*
4. Purpose in life (1–5)	3.20	0.68				−0.37***	−0.26**	0.03	0.31**	−0.16	−0.04	0.07
5. Depressive symptoms (0–15)	4.68	3.13					0.41***	−0.21*	−0.09	0.20	−0.06	−0.24*
6. Self-rated health^b^ (1–5)	3.46	0.71						−0.23*	0.00	−0.03	−0.03	−0.21*
7. Time-to-death (months)	60.71	37.11							−0.19*	−0.15	0.09	0.65***
8. Education (years)	12.46	3.00								−0.13	0.12	0.03
9. Age	90.46	2.81									−0.09	−0.19
10. Sex female	92	78.0%										−0.06
11. Mean number of observations	5.20	4.69										

*Theoretical ranges are provided in brackets*.

a *Higher scores indicate lower pain*.

b *Lower scores indicate better health*.

*p < 0.05; **p < 0.01; ***p < 0.001.

### Measures

#### Pain

Pain was assessed at each of the 16 measurement occasions based on the subscale of the SF-36 (106), comprising two items (“How much bodily pain have you had during the past 4 weeks?” 1 = no pain, 5 = very severe pain; “During the past 4 weeks, how much did pain interfere with your normal work at home?” 1= not at all, 5 = extremely). Following the standard transformation procedure as described in the SF-36 manual (107), both items are combined into one scale which is transformed so that a score range from 0 to 100 results, with higher values indicating lower pain.

#### Eudaimonic Wellbeing

Three subscales of the Ryff Scales of Psychological Wellbeing [PWB; (55)] were assessed at the study's first measurement occasion (T1; “baseline”) and included as indicators of *eudaimonic wellbeing*. Each scale comprises nine items which are answered on a scale ranging from 1 (strongly disagree) to 5 (strongly agree). The PWB components that were included in this study are: Autonomy (e.g., “I have confidence in my opinions, even if they are contrary to the general consensus;” Cronbach's α at baseline = 0.70), Environmental Mastery (e.g., “In general, I feel I am in charge of the situation in which I live;” α = 0.71), and Purpose in Life (e.g., “Some people wander aimlessly through life, but I am not one of them;” α = 0.69).

#### Personality

Neuroticism and extraversion were assessed at T1 by the BFI-K (108), which is a short-form derived from the Big Five Inventory. The scales for neuroticism and extraversion each comprise four items (e.g., for neuroticism: “I worry a lot; for extraversion: “I am outgoing, sociable”) that are answered on a 5-point Likert scale (1= strongly disagree, 5 = strongly agree). A mean score across all four items was computed for each individual. Higher scores indicate higher neuroticism and higher extraversion, respectively (neuroticism α = 0.65; extraversion α = 0.76).

#### Covariates

We controlled for gender, education (years of schooling), subjective health, and depressive symptoms. All covariates were included as time-invariant predictors by using the scores from the first LateLine measurement occasion. Subjective health was assessed based on a single-item question (“How would you rate your general health;” response scale:1 = excellent, 2 = very good, 3 = good, 4 = fair, 5 = poor). Depressive symptoms were assessed based on the 15-item short version of the Geriatric Depression Scale (109). Items (e.g., “Do you think that most people are better off than you are?”) had to be answered with “yes” (1) or “no” (0). A sum score ranging from 0 to 15 was computed, with higher scores indicating more depressive symptoms (Cronbach's α = 0.82).

### Statistical Analyses

Longitudinal multilevel/mixed regression models (110, 111) were computed to investigate time-to-death- and age-related pain trajectories in the oldest-old.

The time unit for both metrics, age and time-to-death, was months, with age grand mean-centered (at 92.8 years, or 1,113.4 months), and time-to-death sign reversed (i.e., −1 indicating 1 month prior to death). Of note, the intraclass correlation coefficient ICC = 0.49 revealed that approximately equal shares of the overall pain variance were due to between-person differences vs. within-person variability. The ICC hence indicates that multilevel modeling is indeed in place to take these two levels of variation into account.

First, we ran basic growth curve models without additional predictors, modeling trajectories of pain, observed in individual *i* at observation *t*, as follows.

Level 1 (within-person) model of age-related trajectories:


(1) $$\begin{array}{l} pain_{ti}=\beta_{0i}+\overset{K}{\underset{k=1}{\sum }}\beta_{ki}{\left(age\right)}_{ti}^{k}+\varepsilon_{ti} \end{array}$$

Level 1 (within-person) model of time-to-death-related trajectories:


(2) $$\begin{array}{l} pain_{ti}=\beta_{0i}+\overset{K}{\underset{k=1}{\sum }}\beta_{ki}{\left(time\text{-}to\text{-}death\right)}_{ti}^{k}+\varepsilon_{ti} \end{array}$$

In both Equations 1, 2, coefficient β_0i_ denotes the random intercept, varying between individuals. In a first step of analyses, linear, quadratic, and cubic trajectories of pain over age or time-to-death were modeled (i.e., in Equations 1, 2, *K* = 1, 2, or 3 for linear, quadratic or cubic growth curves, respectively), with the respective random slope coefficients β_ki_ again varying between individuals. The level 1 (within-person) residual is noted ε_*ti*_ in Equations 1, 2. This first step was focused on model selection, to check for the best fitting curvatures of the age-related and time-to-death-related trajectories, and to compare the fit of the two “competing” time metrics—age vs. time-to-death—in accounting for overall variability (within and between persons) of the pain outcome measure^1^. We compared the model fit of the linear, quadratic vs. cubic trajectory models, as well as of the age vs. time-to-death models with respect to the Bayesian information criterion (BIC) and the proportional reduction in within-person residual variance [*R*^2^, computed according to (114)], which are the criteria commonly used when comparing the model fit of age-related vs. time-to-death-related models [see also (44, 112, 113, 115)]. For model BIC comparison we used the cutoff values as suggested by Kass and Raftery (116), interpreting differences ΔBIC ≥ 1, 3, or 5, respectively, as indication of “positive,” “strong,” or “very strong” evidence in favor of the model with a lower BIC score.

Second, predictors (eudaimonic wellbeing, personality traits and covariates; assessed at the study's first measurement occasion) of both age-related and of time-to-death-related change in pain were analyzed in additional models. Given the above within-person Equations 1, 2, the level 2 equations for the models including the predictors for the random coefficients β_*ki*_ (*k* = 0: intercept; *k* = 1: linear slope) were as follows.


(3) $$\begin{array}{l} \beta_{ki}=\gamma_{k0}+\gamma_{k1}\left(gender_{i}\right)+\gamma_{k2}\left(education_{i}\right)+\\ \;\gamma_{k3}\left({self\text{-}rated\;health}_{i}\right)+\gamma_{k4}\left({depressive\;symptoms}_{i}\right)+\\ \;\gamma_{k5}\left(autonomy_{i}\right)+\gamma_{k6}\left({environmental\;mastery}_{i}\right)+\\ \;\gamma_{k7}\left({purpose\;in\;life}_{i}\right)+\gamma_{k8}\left(neuroticism_{i}\right)+\\ \;\gamma_{k9}\left(extraversion_{i}\right)+\upsilon_{ki} \end{array}$$

These analyses were run with SAS PROC MIXED (117). This procedure accommodates missing due to drop-out and death *via* full information maximum likelihood under the missing at random assumption [MAR; (118)]^2^.

## Results

### Trajectories of Pain Over Age and Time-To-Death

**Figure 1:**
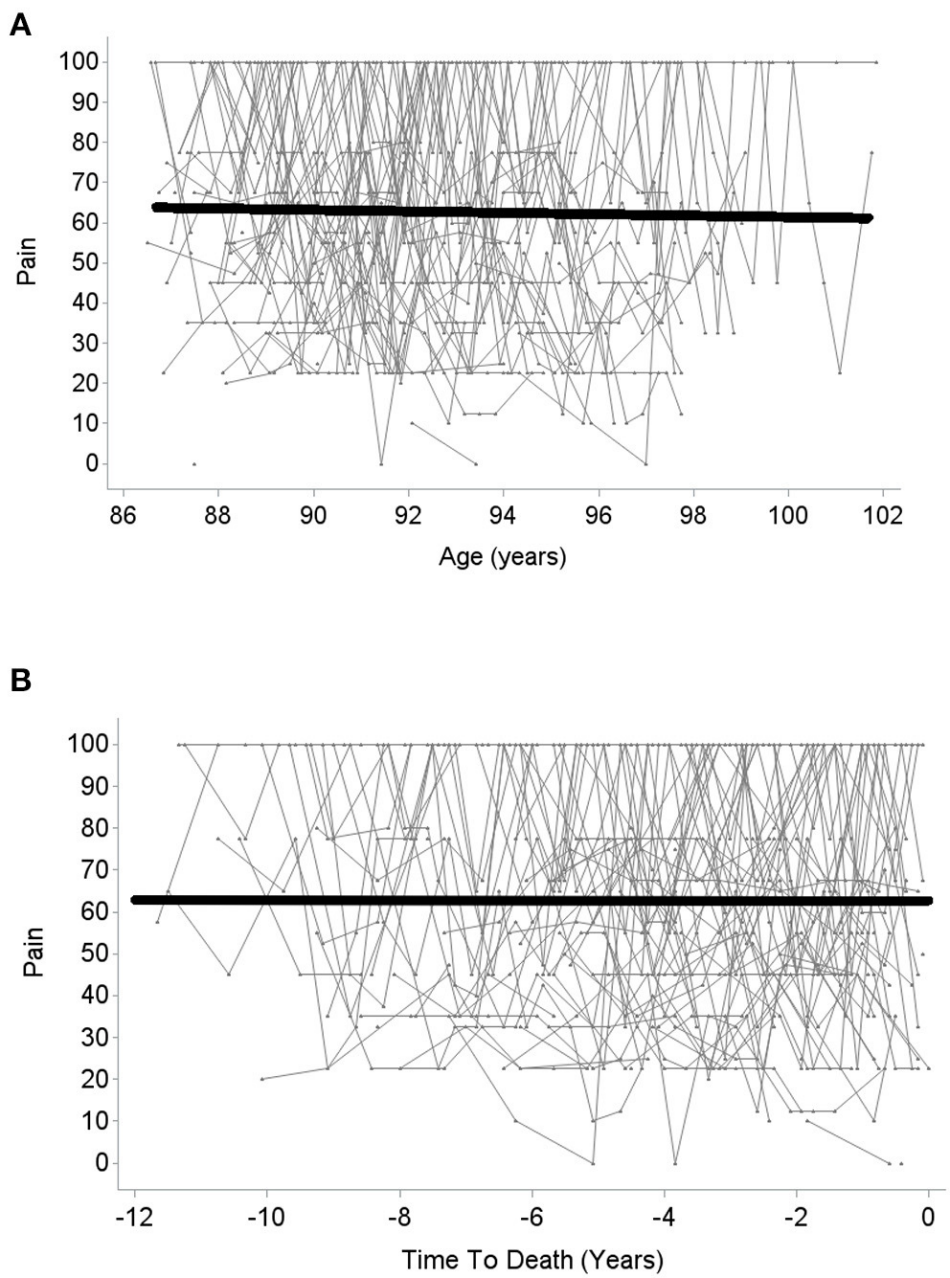
Change in pain over age **(A)** and over time to death **(B)**. Higher scores indicate lower pain.

The quadratic and cubic *age-related change* models did not result in a better model fit than the linear model (BIC_linear_ = 5,593.1, BIC_quadratic_ = 5,597.8, BIC_cubic_ = 5,602.6, there is thus strong/very strong evidence in favor of the linear as compared with the quadratic and cubic models, respectively; *R*^2^ = 0.05 in all models). In the linear model, the fixed slope effect (i.e., the mean-level change) in pain over age was not significant (β_age_ = −0.01, *p* = 0.73; see Figure 1A), but the random variance of the slope component was (see Table 2), indicating substantial interindividual variability in intraindividual within-person pain changes across age. Specifically, inspecting the individual slope estimates, these estimates ranged from −0.26 to +0.33. A negative slope component—indicating increase in pain with advancing age—was estimated for about 61% of the study sample, whereas the slope score was estimated positive for the remaining 39%. There was thus also a substantial proportion of individuals who revealed *less* pain across time, potentially due to higher initial pain which was alleviated or cured by medical or other treatment afterwards. Overall, individual slopes indicating an age-related increase vs. an age-related decrease in pain seem to neutralize each other, resulting in overall mean-level stability of pain over age when averaging all those individual trajectories.

**Table 2:** Longitudinal multilevel regression models of changes in pain over age and over time-to-death.

**Model estimates**	**Pain^a^ change (age)**	**Pain^a^ change (time-to-death)**
**Fixed regression coefficients:**		
Intercept [*SE*]	62.816*** [2.243]	62.729*** [3.137]
Linear slope [*SE*]	−0.015 [0.043]	−0.001 [0.049]
**Random variances:**		
Variance intercept [*SE*]	385.270*** [72.713]	580.160*** [131.220]
Variance linear slope [*SE*]	0.035* [0.019]	0.061* [0.028]
Covariance intercept-slope [*SE*]	0.620 [0.918]	3.636* [1.640]
Residual variance [*SE*]	390.600*** [25.925]	380.840*** [25.223]
BIC	5,593.1	5,588.9
*R* ^2^	0.05	0.08

*Time unit is months. R^2^ was computed according to Xu (114)*.

a *Higher scores indicate lower pain*.

*p < 0.05; ***p < 0.001.

With regard to change over *time-to-death*, again neither the quadratic nor the cubic change model provided a better model fit than linear change model (BIC_linear_ = 5,588.9, BIC_quadratic_ = 5,593.5, BIC_cubic_ = 5,598.2, indicating strong/very strong evidence in favor of the linear as compared with the quadratic and cubic model; linear model *R*^2^ = 0.08, quadratic and cubic model *R*^2^ = 0.07)^3^. In the linear change model, there was—in analogy to change in pain across age—no significant mean-level change (β_time−to−death_ = −0.001, *p* = 0.99; see Figure 1B). However, once again, the random slope variance was significant and indicated large interindividual differences in intraindividual changes. Specifically, individual slope estimates ranged from −0.36 to +0.59. For about 52% of the sample, the estimated slope component was negative, thus indicating an increase in pain with advancing proximity to death, whereas the estimated slope was positive for the remaining 48%. Similar to age-related change in pain, time-to-death-related pain trajectories of increase vs. decline thus neutralized each other, resulting in a pattern of overall mean-level stability in pain over time-to-death.

Comparing the model of change over age with the one of change over time-to-death, the BIC score was in favor of the time-to-death-related change model (BIC = 5,588.9; age-related change model: BIC = 5,593.1; ΔBIC = 4.2, which corresponds to strong evidence for the model with the lower BIC score). Also, the proportional reduction in residual variance was slightly larger in the time-to-death model (time-to-death related model *R*^2^ = 0.08; age-related model *R*^2^ = 0.05). Thus, according to our findings, pain trajectories in the oldest-old can be better described as a function of time-to-death than as a function of chronological age.

### Predictors of Pain Trajectories

**Figure 2:**
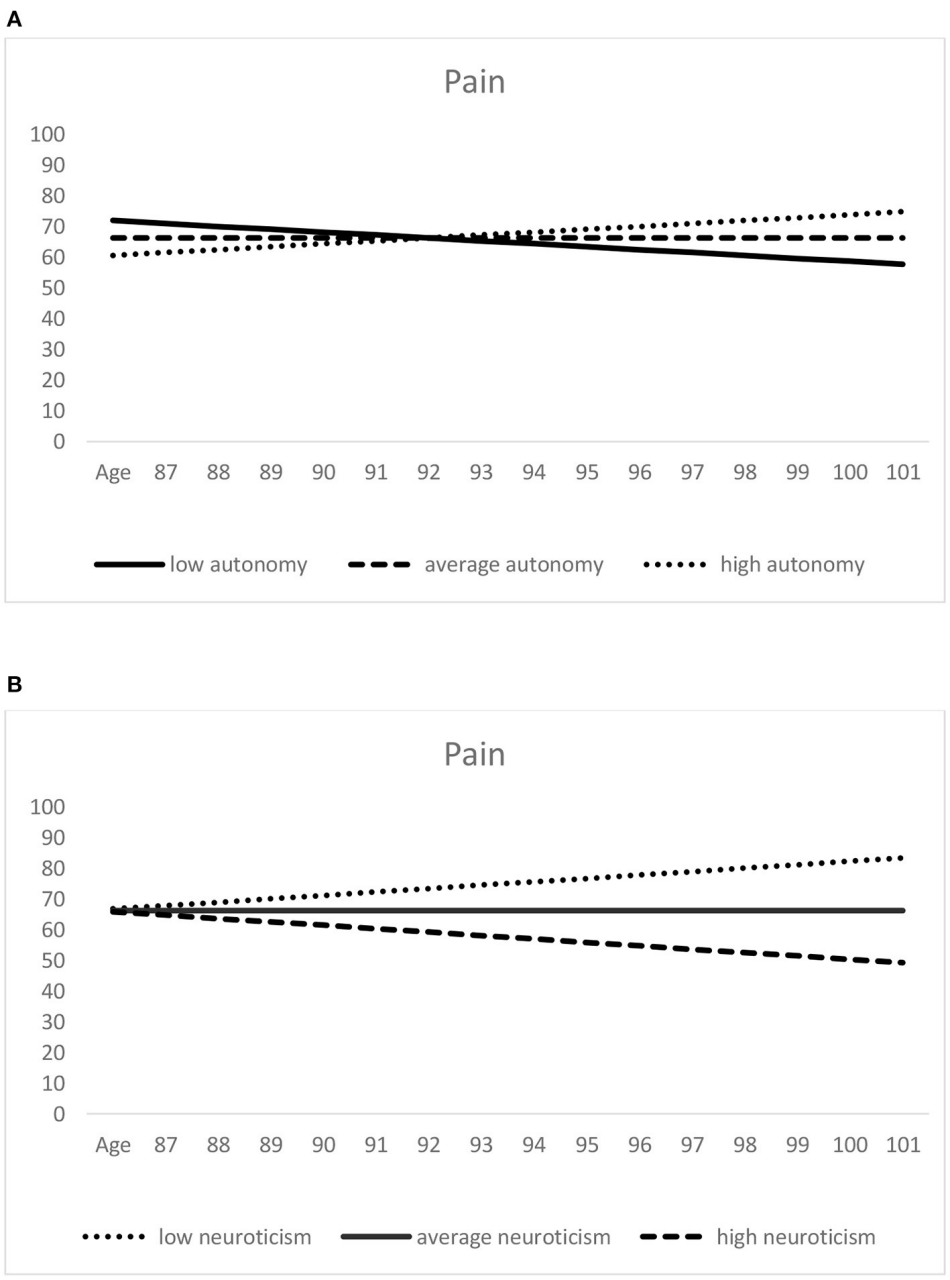
Predictors of age-related pain trajectories: autonomy **(A)** and neuroticism **(B)**. Higher scores indicate lower pain. Low (= one standard deviation below the mean), average (= sample mean score), and high (= one standard deviation above the mean) autonomy/neuroticism were derived from the scores assessed at the first measurement occasion.

When considered over *age* (see Table 3), individual levels of pain (i.e., pain scores at age 92.8 years, the sample grand-mean age) were lower—thus indicating *more* pain—among those with higher neuroticism scores (see Figure 2B). Among the additional covariates, poorer self-rated health was significantly associated with lower SF-36 pain levels (indicating higher pain). Moreover, with respect to the pain slopes, lower autonomy scores and higher neuroticism scores predicted more negative intraindividual change in pain (which indicates a steeper increase or less decrease in pain; see Figure 2).

**Table 3:** Predictors of changes in pain over age and over time-to-death.

**Model estimates**	**Pain^a^ change (age)**	**Pain^a^ change (time-to-death)**
**Fixed regression coefficients:**		
Intercept [*SE*]	66.355*** [4.510]	61.287*** [5.833]
Sex [*SE*]	−2.088 [5.119]	2.802 [6.816]
Education [*SE*]	0.747 [0.721]	0.575 [1.005]
Subjective health [*SE*]	−8.338** [3.112]	−9.443* [4.311]
Depressive symptoms [*SE*]	0.138 [0.920]	2.060 [1.308]
Autonomy [*SE*]	−1.466 [3.921]	6.138 [5.354]
Environmental mastery [*SE*]	4.881 [4.887]	3.310 [6.964]
Purpose in life [*SE*]	2.523 [3.890]	13.106* [5.844]
Extraversion [*SE*]	−3.450 [2.548]	−9.845** [3.538]
Neuroticism [*SE*]	−9.595** [3.207]	−12.424** [4.569]
Linear slope [*SE*]	−0.110 [0.079]	−0.144 [0.084]
Sex*slope [*SE*]	0.082 [0.093]	0.175 [0.100]
Education*slope [*SE*]	0.008 [0.013]	−0.009 [0.014]
Subj. Health*slope [*SE*]	0.037 [0.053]	−0.030 [0.055]
Depr. Symptoms*slope [*SE*]	0.026 [0.018]	0.048* [0.020]
Autonomy*slope [*SE*]	0.141* [0.070]	0.137 [0.072]
Env. Mastery*slope [*SE*]	−0.024 [0.102]	−0.003 [0.103]
Purpose in Life*slope [*SE*]	0.086 [0.078]	0.258** [0.091]
Extraversion*slope [*SE*]	−0.045 [0.046]	−0.168*** [0.050]
Neuroticism*slope [*SE*]	−0.125* [0.062]	−0.077 [0.064]
**Random variances:**		
Variance intercept [*SE*]	237.390*** [51.196]	328.030*** [95.821]
Variance linear slope [*SE*]	0.003 [0.014]	0.013 [0.016]
Covariance intercept-slope [*SE*]	0.859 [0.714]	1.495 [1.060]
Residual variance [*SE*]	396.87*** [27.586]	387.12*** [26.437]
BIC	5,244.5	5,235.7
*R* ^2^	0.04	0.06

*Time unit is months (since 2012). R^2^ was computed according to Xu (114)*.

a *Higher scores indicate lower pain*.

*p < 0.05; **p < 0.01; ***p < 0.001.

**Figure 3:**
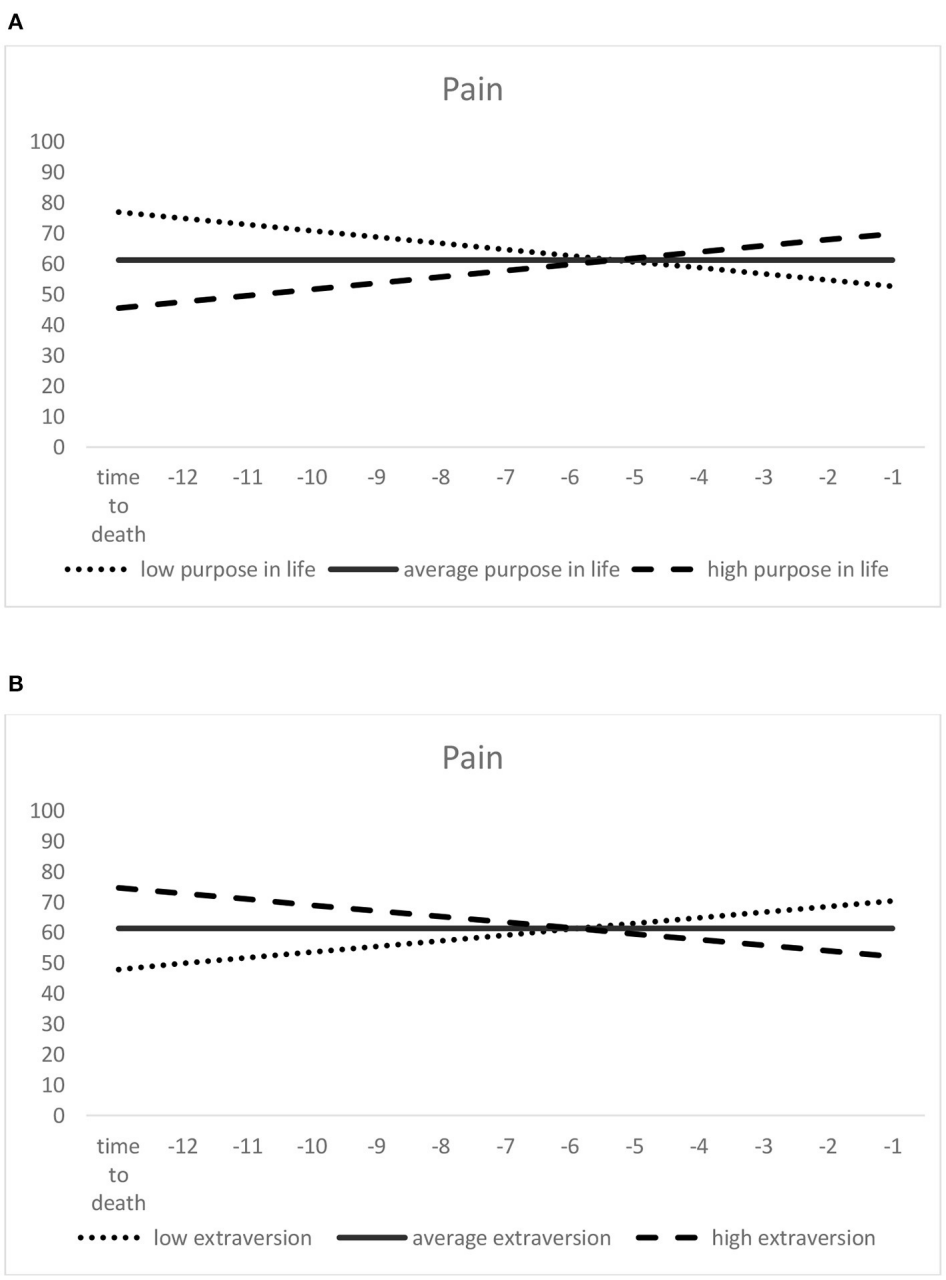
Predictors of time-to-death-related pain trajectories: purpose in life **(A)** and extraversion **(B)**. Higher scores indicate lower pain. Low (= one standard deviation below the mean), average (= sample mean score), and high (= one standard deviation above the mean) purpose in life/extraversion were derived from the scores assessed at the first measurement occasion.

In the model of change in pain over *time to death* (see Table 3), pain scores at the estimated time-to-death were lower (thus indicating greater pain) among those with lower scores on purpose in life (see Figure 3A). They were also significantly lower in those with higher extraversion (see Figure 3B) and higher neuroticism scores, as well as among those with poorer self-rated health. With regard to slope predictors, scoring lower on purpose in life and higher on extraversion predicted more negative pain slopes (indicating a steeper increase or less decrease in pain; see Figure 3). The positive association of higher autonomy with the (time-to-death-related) slope component of pain failed to reach statistical significance (*p* = 0.059). Among the covariates, depressive symptoms were positively associated with within-person pain changes.

## Discussion

In this study, we investigated trajectories of pain among very old adults, taking advantage of an intensive data-collection design that included up to 16 measurement occasions over a 7-year period. As nearly all study participants had deceased in the meantime, we were able to contrast change in pain over chronological age vs. over time-to-death, taking the perspective of primary and tertiary aging (36). Building on theoretical frameworks such as the biopsychosocial model of pain (4, 46) or the disablement process model (47, 48), as well as on available previous empirical research, we examined the role of two sets of psychosocial factors, namely eudaimonic wellbeing as well as personality, for pain trajectories in the oldest-old.

### Pain Trajectories Over Age and Over Time-To-Death

Generally, the mean SF-36 pain scores we observed in our oldest-old study sample were similar to other studies with German samples using the same pain assessment instrument (120, 121). However, these other studies were mostly based on samples that had not yet reached very old age, so that scores are not directly comparable. Interestingly, no significant mean-level change toward more severe pain, neither over chronological age nor over time-to-death, was observed in our study.

There are several potential reasons for this finding of mean-level stability in pain. First, overall stable pain levels might reflect the resilience of very old adults who have outlived many of their peers. Second, individuals with a high susceptibility to pain, e.g., due to terminal disease associated with severe pain load such as cancer, might already have deceased before entering very old age, given that pain is indeed an established risk factor for mortality [e.g., (15, 122)].

Third, subjective or self-reported measures of health and functioning have been found to remain more stable even in very old age and with increasing proximity to death than objective health measures (44), hence supporting a “late-life health paradox” (26). This might explain why pain as a genuinely subjective experience (6), as assessed based on self-reports, remained on average stable both over age and time-to-death.

Fourth, it is important to point out that mean-level stability in pain does by no means indicate that *all* very old adults reveal no change in pain with advancing age or with increasing proximity to death. Rather, we found remarkable heterogeneity both with regard to pain levels at baseline as well as regarding within-person trajectories of pain both over age and time-to-death. Specifically, for more than 60% of the study sample, the estimated change in pain with advancing age indicated a change toward increasing pain. Similarly, for more than 50% of the sample, the estimated change over time-to-death indicated increasing pain. Older and very old adults thus represent a very heterogeneous group (123), not only with regard to levels and changes in domains such as wellbeing or health (22, 103, 124, 125), but also when it comes to the experience of pain. The mean-level stability in pain seems to be to some extent the result of opposing change trends which level each other out, that is increase in pain over time in one group within our sample vs. decrease or stability in another group.

Finally, as also evident from graphical inspection and in line with findings on wellbeing and health in very old age (22, 125), individual pain trajectories do not necessarily follow a systematic—for instance, linear—slope; rather, “ups and downs” in pain are observable across subsequent measurement occasions, which were very dense and only 4 months apart at the end of the study period. It thus seems that there are phases of heightened pain in very old age at some time points which are, however transient (but also recurring in some cases), potentially due to medication or other treatments.

### Psychosocial Predictors of Pain

To some extent, these interindividual differences in within-person pain trajectories were accounted for by psychosocial factors such as wellbeing or personality, which is in line with thereotical conceptions such as the biopsychosocial model of chronic pain (4, 46). Considered over age, higher autonomy levels contributed to less increase, or even steeper decline, of pain over time. Autonomy thus seems to be a crucial eudaimonic wellbeing indicator that might help preventing pain or coping with pain in a way that pain interference in everyday life is minimized. According to self-dermination theory (53), autonomy is—in addition to relatedness and competence—one of the basic and universal psychological human needs. As Ryff (55) states, an individual with a high autonomy score is “self-determining and independent; […]; regulates behavior from within” (p. 45). Deci and Ryan (126) describe autonomy as a characteristic that “refers to volition, to having the experience of choice, to endorsing one's actions at the highest level of reflection” (p. 6). Having the (subjective) experience of choice, and being able to regulate behaviors from within—particularly behaviors to counteract or cope with pain -, could thus be a meaningful resource that buffers negative perceived effects of pain on everyday life.

For time-to-death-related trajectories, another eudaimonic wellbeing component, namely purpose in life, played a significant role. Specifically, less increase—or even steeper decrease—in pain was predicted for those with higher purpose in life scores. A beneficial role of purpose in life for outcomes of health (57) and longevity (56, 58) has been reported before in empirical research, and such beneficial effects seem to persist into very old age and buffer unfavorable pain changes with increasing proximity to death. However, purpose in life was not significantly associated with age-related change in pain. In contrast, autonomy was significantly related only with age-related pain trajectories, whereas its association with time-to-death-related trajectories was only marginally significant. To the extent that differences between the age- vs. time-to-death-related trajectory models might reflect the distinctiveness of primary vs. tertiary aging processes (see footnote 1), the discrepancy in predictor effects between these two models might also indicate that different psychosocial components account for primary vs. tertiary aging processes of pain. Certain wellbeing dimensions—such as purpose in life—might thus gain in importance with regard to health outcomes when it comes to tertiary aging (36, 37), i.e., individuals approaching their end of life. Other dimensions, such as autonomy, predict pain changes solely as part of primary aging, hence as a function of chronological age. Purpose in life is a resource revealing a decreasing trend when people move into old and advanced old age (22, 66, 69, 72); those able to keep this shrinking resource relatively high may be better able to cope with pain in the situation of impending death.

Regarding the effects of personality, different traits predicted age-related vs. time-to-death-related pain trajectories among very old individuals: Whereas, higher neuroticism contributed to changes toward more severe pain with advancing age, worsening of pain over time-to-death was steeper among individuals who were more extraverted at baseline. The role of neuroticism as a health risk factor is well-established [e.g., (77)], so that its association with pain—possibly mediated by use of less adaptive coping strategies (92, 98, 99)—is not surprising. However, the effect of extraversion, generally associated with active coping strategies (92, 98), is—at first glance—unexpected. However, it could be that individuals with a high motivation to seek out social contacts—which is a core constituent of high extraversion—might be particularly frustrated when pain complicates such social activities, so that in consequence, they feel more restricted by pain and report higher pain interference than individuals who are less extraverted and thus less in need of social exchange and stimulation. There is a general debate whether the adaptive or maladaptive character of certain personality traits change when individuals enter very old age. For instance, Mueller et al. (100) discuss that “age- and health-related decreases in agreeableness and extraversion may mirror processes of adaptation, in which no longer attainable social goals (e.g., attending crowded parties in public spaces) are replaced with still attainable ones (e.g., having a small dinner party at home)” (p. 77). According to one study, women with higher extraversion scores reveal a steeper terminal decline in wellbeing (127), which can be interpreted as a maladaptive role of extraversion when it comes to end-of-life trajectories of developmental outcomes. Indeed, our finding with regard to extraversion might imply that lower, rather than greater extraversion in advanced old age is adaptive, at least when outcomes such as pain are considered.

In conclusion, while there is—according to our study findings—no general trend toward greater and more severe pain with increasing age or increasing proximity to death in the oldest-old, there are individuals revealing short-term and transient pain “peaks” at certain measurement occasions, followed by phases of recovery from pain, and there is a substantial proportion of individuals who have a higher risk of experiencing increasing pain levels, both with advancing age and with approaching death. These individuals at risk are those with lower scores on autonomy and on purpose in life, as well as those with higher scores on neuroticism and extraversion.

With regard to practical implications, having identified those psychosocial factors that are related with late-life pain trajectories has the benefit that “in contrast to genetic factors or other non-modifiable environmental factors, psychosocial and psychobehavioral aspects are potentially modifiable variables, making them possible starting points for prevention programs” [(128), p. 23]. Interventions to promote higher eudaimonic wellbeing, particularly autonomy and purpose in life, in advanced old age could thus contribute to preventing, or at least buffering, increasing pain severity and pain interference in this specific life phase. Also, promoting adaptive personality change, e.g., toward greater emotional stability, which seems, according to recent evidence, possible by means of interventions (129), could help to reduce pain in very old age. Lower extraversion was related with more stability in pain over time-to-death, but might not necessarily be adaptive for all developmental outcomes in fourth age, which requires additional investigation.

### Limitations

This study has several strengths and limitations. Regarding strengths of this study, up to 16 measurement occasions—with very dense 4-months assessment intervals in the final study phase—were available, as well as confirmed death dates for 118 of the 124 study participants, thus allowing for in-depth analyses of both age-related and time-to-death-related pain trajectories. Also, the availability of a broad set of eudaimonic wellbeing indicators allowed us to contrast these two broad wellbeing domains and their role for late-life pain by using multiple indicators of eudaimonic wellbeing.

However, there are also several limitations of this study that have to be pointed out. The sample size was rather small, although the remarkable number of repeated observations per individual should provide sufficient statistical power for longitudinal multilevel regression models. Moreover, based on additional analyses using a pattern mixture approach, we did not find that estimates of pain levels or age-related/time-to-death-related pain slopes were different for those with fewer vs. more available repeated observations.

The study sample consisted of individuals living alone, which corresponds to the majority of very old adults (130). However, replication of our findings based on a larger study sample which also comprises individuals not living alone—and individuals living in other areas than the one of our study sample—is desirable.

Moreover, the pain subscale of the SF-36 measures pain severity and pain interference during the past 4 weeks, so that a separation between acute vs. chronic pain is not possible. However, psychosocial predictors of acute vs. chronic pain might not necessarily be the same, which requires further research. Also, only two of the Big Five personality traits were available in this study. Future research should address the role of personality traits beyond neuroticism and extraversion—particularly of conscientiousness as a highly health-relevant personality trait (131), but also of openness for experience, which might be helpful for coping with pain—for late-life pain trajectories. Moreover, a short scale was used to assess neuroticism and extraversion, with four items per trait, so that the role of trait facets for pain could not be investigated in this study. Psychometric properties (Cronbach's α) of the short scales were not optimal, particularly for neuroticism, so that replication based on more comprehensive personality assessment instruments is needed. Use of medication (analgesics etc.) might have an impact on pain in the oldest-old and also shape pain trajectories over time and within-person variability in pain, but—given the psychological scope of this research project—medication had not been assessed in this study.

Mediators linking psychosocial predictors (wellbeing and personality) to pain trajectories in very old age were not investigated in this study. Future research should identify such mediating pathways as well as moderators of associations between psychosocial functioning and pain in advanced old age.

Finally, a general challenge of research addressing time-to-death-related changes is to collect data from individuals when they are close to death—and in many cases no longer willing or able to take part in empirical studies. In our study sample, only about 10% of all data points were collected during individuals‘ last year of life, so that changes in pain in the very last months of life might not have been detected by our approach. Additionally, the age range of our study sample was restricted to very old age, so that potential increases in pain from early-old age to old age could not be identified based on our study sample.

## Conclusion

In this study, we investigated trajectories of pain in the oldest-old, both over primary aging/chronological age (time since birth) and over tertiary aging/time-to-death. While there was no significant mean-level change in pain over age or time-to-death, baseline pain and within-person pain changes revealed a remarkable heterogeneity. Additionally, we observed a substantial proportion of within-person variability from one measurement occasion to the next that does not necessarily follow a systematic—linear or non-linear—function. Among the psychosocial predictors that were significantly associated with pain trajectories, higher autonomy scores were predictive of less increase—or oven greater decrease—in pain, when considered over age. Higher purpose in life was associated with less steep increase, or greater decline, in pain over time-to-death. Higher neuroticism was associated with age-related change toward more severe pain, whereas higher extraversion predicted a steeper change toward more severe pain over time-to-death. In conclusion, different psychosocial factors seem to predict age-related and time-to-death-related change in pain among oldest-old individuals. Promoting eudaimonic wellbeing—particularly autonomy and purpose in life—in very old age might contribute to preventing pain, or at least to buffering the negative consequences of pain on everyday life, in this life phase.

## Data Availability Statement

The raw data supporting the conclusions of this article will be made available by the authors, without undue reservation.

## Ethics Statement

The studies involving human participants were reviewed and approved by Ethics Commission, Faculty of Behavioral and Cultural Studies, Heidelberg University. The patients/participants provided their written informed consent to participate in this study.

## Author Contributions

MW computed all statistical analyses and wrote the results section. MW, OS, and H-WW conceptualized the study (introduction section), wrote the discussion section, and were involved in writing all other parts of the manuscript. All authors contributed to the article and approved the submitted version.

## Funding

The LateLine Study used in this research was funded by the German Research Foundation (SCHI 1024/3–1 and SCHI 1024/3–2), as well as by the Network Aging Research (NAR) of Heidelberg University.

## Conflict of Interest

The authors declare that the research was conducted in the absence of any commercial or financial relationships that could be construed as a potential conflict of interest.

## Publisher's Note

All claims expressed in this article are solely those of the authors and do not necessarily represent those of their affiliated organizations, or those of the publisher, the editors and the reviewers. Any product that may be evaluated in this article, or claim that may be made by its manufacturer, is not guaranteed or endorsed by the publisher.

## Footnotes

^1^Note that the models denoted by Equations 1, 2 “fully” adjust the longitudinal pain measures to chronological age vs. time-to-death, without controlling for each other, respectively. Therefore, considering these equations as statistical models of primary vs. tertiary aging processes unfolding in pain, it should be kept in mind that these processes (and also secondary aging) may co-occur (112, 113) and that in particular the overlay of age- and time-to-death-related effects in intra-individual changes is not strictly disentangled in either of both models. We did not run a model simultaneously including both time metrics for reasons of model parsimony, due to the narrow between-person sample size as well as the very restricted (baseline) age range of this oldest-old study sample. However, the comparison of model fit achieved with Equations 1 vs. 2 may be tentatively considered in terms of the relative strength of primary vs. tertiary aging processes, as revealed by the overall predictive effect of each time metric in accounting for pain variance in this specific very old study sample close to death.

^2^Maximum likelihood estimation includes missing-at-random (MAR) treatment in estimating the intercepts and slopes of those who dropped out early and were thus measured at only few (<3) occasions. However, if the MAR assumption is not met [i.e., missing-not-at-random; MNAR, e.g., (118)], the intercept and slope estimation may be biased. We therefore applied a pattern mixture approach [for details, (119)] as follows: Dividing the study sample into individuals with only one or two observations (“early dropouts”; about 45% of the study sample) and those with more than two observations (“late dropouts” and non-dropouts), we used this dropout-group division as (level 2) predictor of intercept and slope in the (linear) age- or time-to-death trajectories. The differentiation between early and late dropouts did not reveal any significant effects. The estimates of pain trajectories did thus not significantly vary as a function of the number of available repeated observations, suggesting that the MAR estimates of pain intercepts and slopes may be trustworthy.

^3^We also checked for exponential growth functions using the SAS PROC NLMIXED command (117). However, model fit in terms of BIC was not better in exponential models compared to models of linear change, both over age and over time-to-death. Also, the estimates of the fixed effects of the change components within the exponential growth functions were not significant, indicating – in line with the models of linear change – mean-level stability of pain, both over age and time-to-death.
